# Spontaneous Rectus Sheath Hematoma Mimicking Bladder Tamponade: A Case Report

**DOI:** 10.3390/jcm15176623

**Published:** 2026-08-27

**Authors:** Yupeng Wang, Jingyu You, Runzhe Wang, Yeqing Mao, Xiaoyi Chen, Baiye Jin, Guanghou Fu

**Affiliations:** 1 Department of Urology, The First Affiliated Hospital, School of Medicine, Zhejiang University, Hangzhou 310009, China; 22518253@zju.edu.cn (Y.W.); 22318564@zju.edu.cn (R.W.); 1313019@zju.edu.cn (Y.M.); 11818376@zju.edu.cn (X.C.); 2 Zhejiang Engineering Research Center for Urinary Bladder Carcinoma Innovation Diagnosis and Treatment, Hangzhou 310024, China; 3 Department of Critical Care Medicine, The First Affiliated Hospital, School of Medicine, Zhejiang University, Hangzhou 310003, China; 21718172@zju.edu.cn

**Keywords:** spontaneous rectus sheath hematoma, bladder tamponade, case report

## Abstract

**Background:** Spontaneous rectus sheath hematoma is a known but relatively rare complication of anticoagulant therapy. Because its clinical features are similar to various intra-abdominal pathologies, it is often misdiagnosed as other types of acute abdomen in emergency settings. **Case Presentation:** The patient presented with acute lower abdominal pain, suprapubic distension, and sudden anuria following anticoagulation therapy. Abdominal computed tomography revealed a giant pelvic mass mimicking a distended bladder, suggestive of bladder tamponade. A percutaneous cystostomy was then performed, which, however, drained a large volume of non-clotted blood rather than blood clots. Subsequent radiological and clinical evaluation confirmed a giant spontaneous rectus sheath hematoma. Given the patient’s critical condition and poor prognosis, after thorough discussion with the family, the decision was made to transfer the patient to a local hospital for continued supportive care. **Conclusions:** This case highlights the diagnostic challenges of giant or atypically located rectus sheath hematomas, which may clinically and radiologically mimic intra-abdominal or urological emergencies such as bladder tamponade. Conventional clinical evaluation and erroneous radiological interpretation can lead to a misdiagnosis, occasionally resulting in inappropriate invasive interventions. Recognizing specific radiological signs, such as internal fluid layering and extravesical catheter placement, combined with multiplanar computed tomography reconstruction, helps minimize diagnostic pitfalls, prevent unnecessary procedures, and optimize clinical outcomes.

## 1. Introduction

Spontaneous rectus sheath hematoma is a relatively rare but potentially life-threatening complication of anticoagulation therapy, resulting from bleeding into the rectus sheath, after damage to the epigastric arteries or by muscular tear [1]. With the increasingly widespread use of anticoagulant and antiplatelet agents, its incidence has been steadily rising, making early diagnosis critical [2,3].

The most common symptoms and signs of spontaneous rectus sheath hematoma are abdominal pain and a palpable abdominal mass. Associated symptoms may include nausea, vomiting, fever, and urinary retention [1,4]. Due to its nonspecific clinical presentation, the initial diagnosis of spontaneous rectus sheath hematoma is challenging and requires a broad differential diagnosis to exclude many other abdominal pathologies. Imaging, especially computed tomography (CT), with a high sensitivity and specificity, is crucial for diagnosis [4,5].

However, when the hematoma is particularly large or in an atypical location, its clinical presentation may lead clinicians to misinterpret it as bladder tamponade on CT imaging. In this case, we report a spontaneous rectus sheath hematoma that was initially misdiagnosed as bladder tamponade, resulting in an unnecessary cystostomy.

## 2. Case Presentation

### 2.1. Clinical History and Presentation

An 84-year-old woman was transferred to the intensive care unit (ICU) of our hospital because of acute respiratory failure requiring mechanical ventilation for 1 day, which occurred 6 days after surgery for a left intertrochanteric femoral fracture.

The patient had a past medical history of multiple chronic conditions, including hypertension, diabetes mellitus, coronary atherosclerotic heart disease, chronic nonvalvular atrial fibrillation, and chronic cardiac insufficiency, but no history of abdominal wall trauma or surgery. The patient had been taking rivaroxaban (1 tablet daily) regularly for long-term anticoagulation, along with other corresponding medications for the chronic diseases.

Two weeks before admission, the patient presented to a local hospital with left hip swelling and pain after a fall. Imaging confirmed a left intertrochanteric comminuted femoral fracture, and the patient was admitted to the orthopedic department. Rivaroxaban was discontinued upon admission. Seven days after admission, the patient underwent closed reduction and intramedullary nailing of the left femoral fracture. On the third postoperative day, the patient developed agitation, delirium, oliguria, and progressive decline in oxygenation. Chest CT showed localized atelectasis and a small amount of bilateral pleural effusion. Arterial blood gas analysis indicated respiratory failure and metabolic acidosis (blood lactate 2.6 mmol/L), and renal function tests indicated acute kidney injury (serum creatinine 243 μmol/L). The patient was subsequently transferred to the ICU, where emergency endotracheal intubation with mechanical ventilation was performed, along with bronchoalveolar lavage under fiberoptic bronchoscopy. After the above treatments, the patient’s condition improved somewhat.

**Figure 1:**
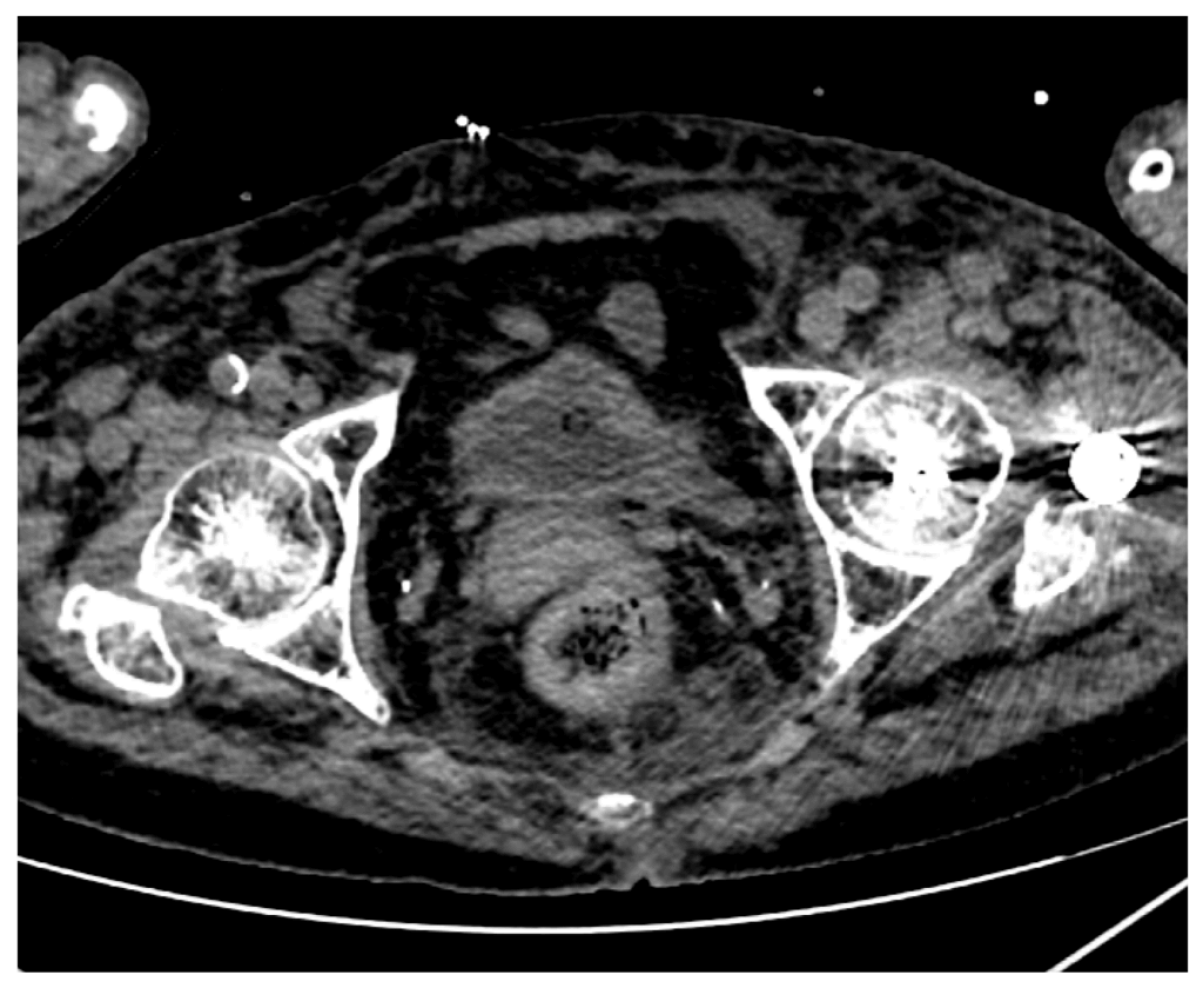
Non-contrast CT of the whole abdomen on admission showed no evidence of intra-abdominal hemorrhage or hematoma.

For further treatment, the patient was transferred to our hospital. On admission, the patient was sedated and on mechanical ventilation, with stable vital signs. A non-contrast CT of the entire abdomen performed on the day of admission showed no evidence of intra-abdominal hemorrhage or hematoma (Figure 1). Given the patient’s history of atrial fibrillation and prolonged bed rest, a therapeutic-dose anticoagulation regimen was promptly initiated to prevent stroke and venous thromboembolism: enoxaparin 4000 IU was administered subcutaneously once daily.

### 2.2. Clinical Progression and Initial Diagnosis

After admission, the patient received active comprehensive symptomatic and supportive treatment, including anti-infective therapy, inotropic and diuretic agents, expectorants, antispasmodics, as well as sedation and analgesia. The patient’s condition subsequently improved, with complete blood count parameters, electrolytes, and infection markers gradually stabilizing. On the third day of admission, the patient was successfully extubated. At that time, the body temperature was 37.2 °C, blood pressure was 113/62 mmHg, hemoglobin was 76 g/L, whole-blood lactate was 1.5 mmol/L, and 24 h urine output was 1500 mL. After extubation, because follow-up pulmonary artery CT angiography suggested possible thromboembolism in small branches and the D-dimer level was elevated, the anticoagulation regimen was adjusted to intensified anticoagulation with enoxaparin sodium injection 4000 IU administered subcutaneously every 12 h.

On day 4 of anticoagulation therapy, the patient’s condition deteriorated. She repeatedly complained of severe lower abdominal pain, and physical examination revealed marked suprapubic distension and lower abdominal tenderness. Meanwhile, the urine output via the urinary catheter gradually decreased and then dropped sharply until anuria developed.

**Figure 2:**
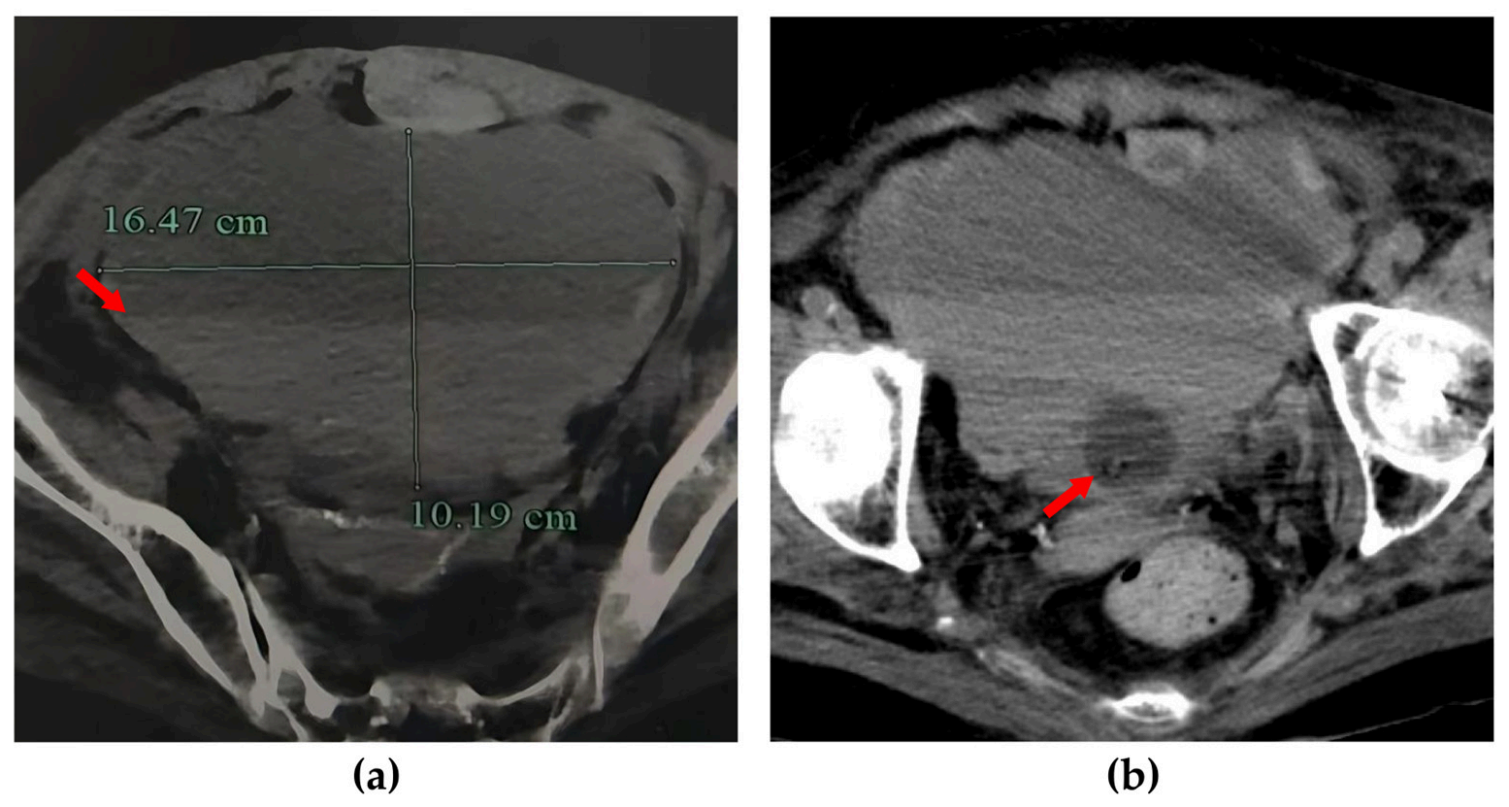
Non-contrast abdominal CT. (**a**) A giant, bladder-shaped rectus sheath hematoma with an internal fluid-fluid level (arrow). (**b**) The urinary catheter (arrow) remains outside the fluid-filled mass without entering its cavity.

Given the presentation of severe abdominal pain, suprapubic distension, and anuria, the clinical team initially considered a urological emergency. Consequently, in light of the patient’s anticoagulation therapy, the pelvic mass identified on urgent abdominal CT and bedside ultrasound was initially interpreted as bladder tamponade secondary to spontaneous bladder hemorrhage (Figure 2).

### 2.3. Urgent Intervention and Diagnostic Correction

**Figure 3:**
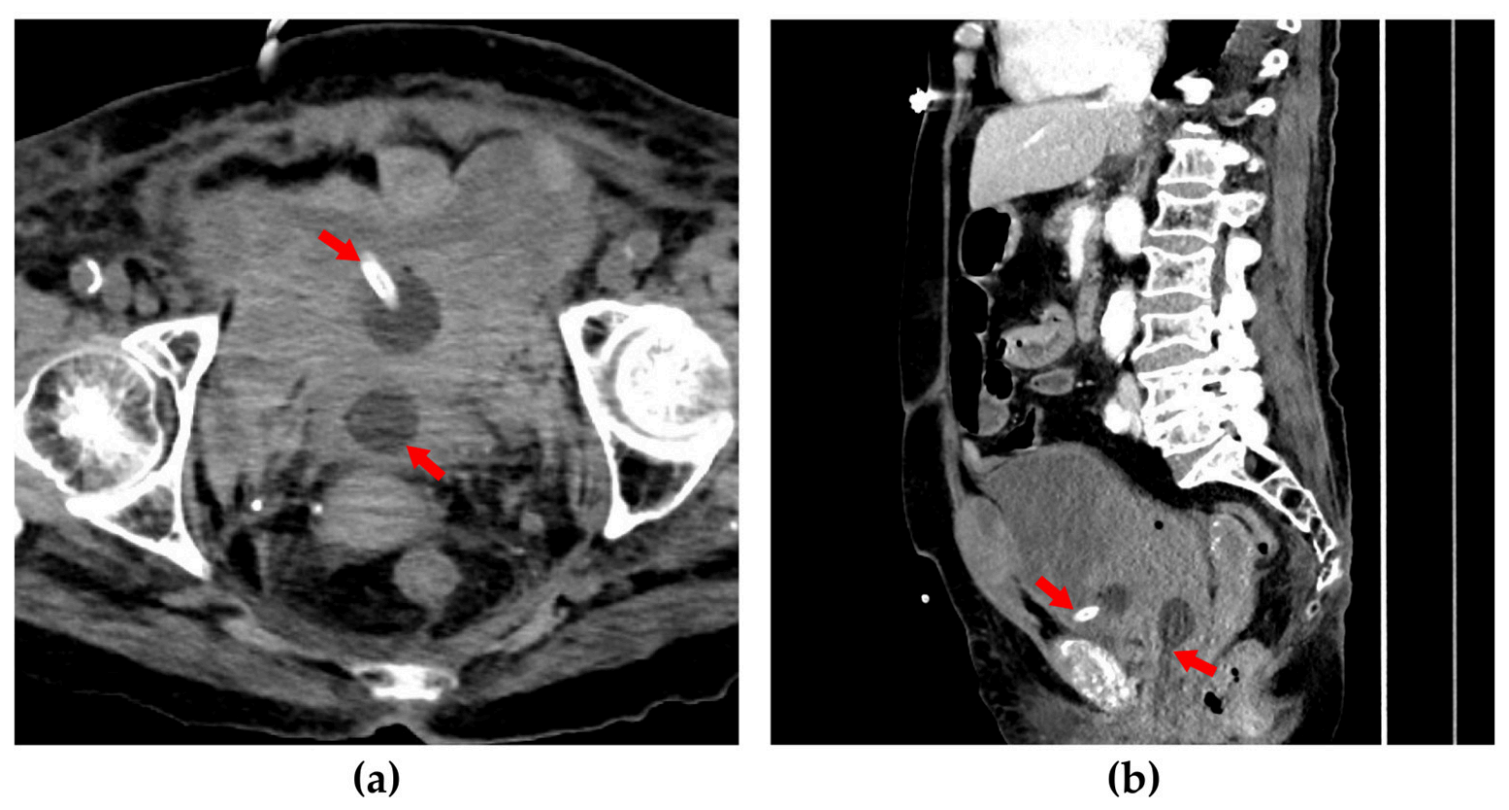
CT urography in (**a**) axial and (**b**) sagittal views. The upper red arrow indicates the cystostomy tube, and the lower red arrow points to the urinary catheter.

After urology consultation, attempts were first made to replace the urinary catheter, but no urine was obtained. Bladder irrigation was then attempted, but fluid could neither be instilled nor aspirated. Based on the diagnosis of bladder tamponade, percutaneous cystostomy was deemed necessary to relieve the suspected obstruction. However, immediately after placement, the cystostomy tube drained a large amount of non-clotted blood, while the urinary catheter drained clear urine. These findings suggested that the cystostomy tube had entered the hematoma cavity rather than the bladder. A follow-up CT urography combined with multiplanar reconstruction clearly demonstrated that the tip of the cystostomy tube was located within the hematoma cavity, whereas the urinary catheter was correctly positioned in the bladder (Figure 3). This provided definitive radiological evidence that the procedure had inadvertently entered a rectus sheath hematoma.

### 2.4. Treatment Outcomes

After the cystostomy tube was inadvertently placed into the cavity of a rectus sheath hematoma, approximately 500 mL of non-clotted blood was immediately drained through the tube. A repeat complete blood count showed that hemoglobin (Hb) had dropped sharply to 49 g/L. The medical team immediately initiated aggressive resuscitation, urgently administering fluid volume expansion and transfusing 2 units of leukocyte-depleted packed red blood cells. Concurrently, all anticoagulant medications were discontinued due to the high risk of active bleeding. After these emergency measures, the patient’s vital signs temporarily stabilized.

Over the following three days, the patient’s Hb dropped to a nadir of 46 g/L, accompanied by transient hypotension (89/53 mmHg). The medical team continued aggressive fluid resuscitation and administered an additional 3.5 units of leukocyte-depleted packed red blood cells along with 240 mL of fresh frozen plasma. After treatment, the patient’s Hb stopped declining, rebounded, and stabilized at approximately 50–53 g/L, while blood pressure stabilized at around 110/60 mmHg. During this period, the bloody drainage from the cystostomy tube gradually decreased, indicating that active bleeding had been controlled.

However, on the fourth postoperative day, follow-up color Doppler ultrasound of the bilateral lower extremity vessels revealed newly developed intermuscular venous thrombosis. This clinical finding posed an extremely severe therapeutic dilemma: restarting anticoagulation would expose the patient to a high risk of fatal rebleeding, whereas continuing to withhold anticoagulation would carry the risk of worsening thromboembolism. Meanwhile, although the infection markers had improved compared with previous values, the patient’s respiratory function progressively deteriorated, with the oxygenation index declining to approximately 250 mmHg under high-flow nasal cannula humidified oxygen therapy.

Faced with persistent respiratory dysfunction and the dilemma between hemostasis and anticoagulation, the medical team informed the family of the possibility of further deterioration and the potential need for reintubation and mechanical ventilation. After thorough communication, the family declined further invasive resuscitation measures and requested transfer to a local hospital for conservative supportive care. After transfer, the patient’s Hb steadily recovered, but the respiratory failure did not improve. Subsequently, the patient was transferred to a local township health center for hospice care and eventually passed away.

### 2.5. Timeline of Clinical Course

To provide a clear and structured overview of the patient’s disease progression and management, the key clinical events and detailed timeline of the patient’s care are summarized in Table 1.

## 3. Discussion

Spontaneous rectus sheath hematoma, caused by hemorrhage from rupture of one of the epigastric arteries or from a tear of the muscle itself, is a rare clinical event. Its main risk factors include trauma, a history of abdominal surgery, and anticoagulant therapy, with the use of anticoagulants potentially being the most significant [1]. It has been reported that nearly 80% of patients with spontaneous rectus sheath hematoma are receiving some form of anticoagulation. Additionally, it can occur in other conditions such as pregnancy, severe coughing, hypertension, or atherosclerosis [6,7,8].

The most common symptoms and signs of spontaneous rectus sheath hematoma are abdominal pain and a palpable abdominal mass. For large spontaneous rectus sheath hematomas, patients may present with signs and symptoms of hemodynamic instability [9,10]. They may also compress the bladder, causing urinary obstruction and subsequent retention [9,11]. Given its nonspecific presentation, spontaneous rectus sheath hematoma is often misdiagnosed as other causes of acute abdomen, such as appendicitis, cholecystitis, diverticulitis, incarcerated hernia, or urinary tract obstruction.

Ultrasonography is employed as a first-line test in the evaluation of acute abdominal pain due to its cost-effectiveness, safety, and ease of use [12,13]. However, its diagnostic accuracy for spontaneous rectus sheath hematoma is operator-dependent. CT imaging has been reported to have a sensitivity and specificity approaching 100% for spontaneous rectus sheath hematoma and is the diagnostic modality of choice [5,14]. However, under the time pressure of an acute presentation, clinicians who are potentially anchored by the clinical presentation might overlook critical radiological features, leading to erroneous clinical decisions.

In this case, the initial diagnostic approach was strongly influenced by anchoring bias, whereby the sudden onset of lower abdominal pain, abdominal distension, and anuria was prematurely attributed to bladder tamponade [15]. In fact, the anuria in this patient was not caused by intrinsic renal failure or intravesical obstruction, but by severe mechanical compression of the bladder and urethral outlet by the large extramural hematoma. Only after the hematoma cavity was inadvertently punctured and blood was drained did the compression resolve, allowing the accumulated clear urine to be excreted smoothly. Concurrently, this cognitive bias prevented the team from conducting a thorough analysis of the imaging details, ultimately leading to invasive procedures that could have been avoided. Retrospective review revealed two overlooked key imaging features that constituted important diagnostic clues and should have raised suspicion for a rectus sheath hematoma.

First, both the CT scan and the bedside ultrasound clearly demonstrated a fluid-fluid level within the pelvic mass. This is a classic sign of a subacute or chronic hematoma, in which settled red blood cells separate from the overlying serum within a relatively enclosed and stable cavity [16]. In contrast, acute bladder tamponade typically presents with irregular blood clots filling the bladder lumen, appearing as a heterogeneous continuous structure, and rarely exhibits such a distinct and stable horizontal layering [15].

Second, CT imaging clearly demonstrated that the inflated balloon of the urinary catheter remained outside this fluid-filled mass and did not enter its cavity. This finding provides strong evidence that the mass was an extravesical lesion separate from the bladder, rather than a distended bladder, which was further confirmed by multiplanar CT reconstruction.

Previous literature has described such potentially misleading findings as the “pseudobladder sign,” yet clear differential diagnostic criteria have not been established [17]. Through this case, we emphasize that in patients receiving anticoagulant therapy without a history of abdominal trauma or surgery, the sudden onset of acute abdominal pain accompanied by a pelvic mass and anuria should raise high clinical suspicion for spontaneous rectus sheath hematoma. On axial non-contrast CT of the entire abdomen, identification that the urinary catheter balloon failed to enter the lesion provides a critical diagnostic clue, whereas internal layering within the collection serves as a supportive finding. However, as these axial signs alone may lack sufficient specificity, multiplanar CT reconstructions should be performed to carefully evaluate the anatomical relationship of the mass with the rectus sheath and the bladder, thereby enabling accurate differentiation and helping to avoid both misdiagnosis and unnecessary invasive procedures. In this case, CT urography performed following cystostomy, coupled with multiplanar reconstructions, effectively confirmed that the lesion was extravesical and originated from the rectus sheath, thereby establishing the correct diagnosis.

In addition to bladder tamponade, various lower abdominal lesions can also present with lower abdominal pain, abdominal distension, and anuria, and are easily confused with spontaneous rectus sheath hematoma. These mainly include extraperitoneal bladder rupture, urinoma, retroperitoneal hematoma, and large ovarian or pelvic tumors.

Extraperitoneal bladder rupture and urinoma usually have a clear history of trauma or iatrogenic injury [18]. The massive fluid accumulation they produce can compress and displace the bladder, leading to acute urinary retention. CT cystography demonstrating extravasation of contrast medium can confirm the extraperitoneal bladder rupture, while delayed contrast-enhanced CT may be the optimal diagnostic modality for urinoma [19,20].

Retroperitoneal hematoma is often secondary to trauma, vascular interventional procedures, or anticoagulant therapy [21]. Although its clinical manifestations overlap with those of spontaneous rectus sheath hematoma, the hematoma is usually located deeper. On physical examination, Carnett’s sign and Fothergill’s sign are typically negative, which helps differentiate it from abdominal wall-origin rectus sheath hematoma [1].

Large gynecologic lesions or pelvic tumors mainly cause obstruction through mass effect compressing the urethral outlet. Multiplanar CT reconstruction can effectively differentiate them by tracing the origin of the lesion and its relationship to the abdominal wall muscles.

In summary, differentiation of spontaneous rectus sheath hematoma from the above lesions requires comprehensive consideration of the patient’s history, laboratory findings, and imaging features. Among these, analysis of anatomical spaces and structures based on multiplanar CT reconstruction plays a decisive role in establishing the final diagnosis and can significantly reduce unnecessary surgical exploration.

The management of spontaneous rectus sheath hematoma primarily depends on the patient’s hemodynamic stability and the characteristics of the hematoma. In the vast majority of cases, non-expanding hematomas in stable patients are self-limiting and can be successfully managed conservatively [22,23]. In contrast, invasive interventions, such as selective embolization or surgical ligation, are reserved only for cases of hemodynamic instability that are unresponsive to fluid resuscitation, or for those with signs of an expanding hematoma or abdominal compartment syndrome [24,25]. In this case, selective embolization was not performed because active bleeding was temporarily controlled after immediate discontinuation of anticoagulants and aggressive fluid resuscitation with blood transfusion, as evidenced by a progressive decrease in bloody drainage from the cystostomy tube and stabilization of hemoglobin levels and blood pressure. If the patient’s condition fails to improve after these initial measures and the overall clinical status permits, CT angiography should be considered to clearly identify the bleeding site, followed by selective embolization.

Simple percutaneous drainage of the hematoma alone is not recommended, as it may not only introduce a risk of infection but also potentially disrupt the stable hematoma cavity, leading to renewed bleeding and thereby increasing the risk of hemorrhage aggravation. Therefore, in this case, the percutaneous drainage performed due to misdiagnosis was fundamentally inappropriate for managing the spontaneous rectus sheath hematoma. It not only failed to address the underlying cause of bleeding but may also have introduced new risks by potentially disrupting the hematoma cavity.

## 4. Conclusions

This case of an 84-year-old woman with enoxaparin-associated spontaneous rectus sheath hematoma mimicking bladder tamponade highlights the critical diagnostic pitfalls that can arise in complex acute settings. By sharing this uncommon case, we aim to increase awareness of giant extravesical hematomas that present as urological emergencies and to emphasize the importance of avoiding diagnostic anchoring bias to prevent inadvertent invasive procedures. Clear identification of a separate urinary bladder containing the urinary catheter balloon, combined with multiplanar CT reconstruction to delineate the anatomical relationships between the mass and adjacent structures, was essential for accurate differential diagnosis, whereas internal fluid layering served as a supportive sign. However, definitive diagnostic criteria cannot be established from a single case report, and clinicians should maintain heightened vigilance when evaluating atypical pelvic masses in similar clinical scenarios.

## Figures and Tables

**Table 1:** CARE Timeline of Clinical Course.

Time	Clinical Event
Past history	The patient was an 84-year-old female who had a history of hypertension, diabetes, coronary heart disease, chronic atrial fibrillation, and cardiac insufficiency, and was on long-term rivaroxaban therapy.
2 weeks before admission	She fell and suffered a left intertrochanteric femoral fracture. She was admitted to a local hospital, and her rivaroxaban treatment was discontinued.
1 week before admission	She underwent closed reduction and intramedullary nailing of the left femoral fracture.
3 days before admission	She developed agitation, delirium, oliguria, acute respiratory failure, and acute kidney injury. Consequently, she was transferred to the intensive care unit for endotracheal intubation and mechanical ventilation.
Day 1 of admission	The patient was transferred to our intensive care unit due to acute respiratory failure. A baseline non-contrast abdominal computed tomography scan showed no evidence of abdominal hemorrhage. Subcutaneous enoxaparin therapy at a dose of 4000 IU daily was initiated.
Day 3 of admission	She was successfully extubated. A pulmonary computed tomography angiography suggested small-branch pulmonary thromboembolism. Therefore, her anticoagulation regimen was intensified to subcutaneous enoxaparin 4000 IU every 12 h.
Day 4 of admission	The patient developed severe lower abdominal pain, suprapubic distension, and sudden anuria. An emergency non-contrast abdominal CT revealed a giant pelvic mass, which was initially misdiagnosed as bladder tamponade.
Urgent intervention	A percutaneous cystostomy was performed, which drained approximately 500 mL of non-clotted blood. Her hemoglobin level dropped sharply to 49 g/L. Emergency fluid resuscitation and blood transfusion with 2 units of red blood cells were administered, and anticoagulation therapy was discontinued.
Diagnostic correction	A follow-up computed tomography urography with multiplanar reconstruction confirmed that the lesion was a giant spontaneous rectus sheath hematoma. The imaging also demonstrated that the cystostomy tube had been misplaced into the hematoma cavity.
Days 1–3 post-procedure	Her hemoglobin level reached its nadir at 46 g/L, accompanied by transient hypotension. She was managed with aggressive resuscitation, including transfusion of 3.5 units of red blood cells and 240 mL of fresh frozen plasma. Active bleeding was controlled, and her hemoglobin level stabilized at 50–53 g/L.
Day 4 post-procedure	Ultrasonography detected deep venous thrombosis in her lower extremities. Meanwhile, her respiratory function deteriorated with an oxygenation index of approximately 250 mmHg. Faced with a severe clinical dilemma between maintaining hemostasis and resuming anticoagulation, her family declined further invasive resuscitation.
Outcome	The patient was transferred to a local hospital for conservative supportive treatment. Although her hemoglobin level gradually recovered, her respiratory failure progressed, and she eventually passed away.

## Data Availability

The original contributions presented in the study are included in the article; further inquiries can be directed to the corresponding author.

## Abbreviations

The following abbreviations are used in this manuscript:

CT	Computed Tomography
ICU	Intensive Care Unit
Hb	Hemoglobin
